# Modified Urethral Graciloplasty Cross-Innervated by the Pudendal Nerve for Postprostatectomy Urinary Incontinence: Cadaveric Simulation Surgery and a Clinical Case Report

**DOI:** 10.1055/a-1995-1513

**Published:** 2023-10-17

**Authors:** Hisashi Sakuma, Masaki Yazawa, Makoto Hikosaka, Yumiko Uchikawa-Tani, Masayoshi Takayama, Kazuo Kishi

**Affiliations:** 1 Department of Plastic and Reconstructive Surgery, Ichikawa General Hospital, Tokyo Dental College, Tokyo, Japan; 2 Department of Plastic and Reconstructive Surgery, Yokohama Municipal Citizen's Hospital, Yokohama, Japan; 3 Department of Plastic and Reconstructive Surgery, Keio University School of Medicine, Tokyo, Japan; 4 Department of Plastic and Reconstructive Surgery, National Center for Child Health and Development, Tokyo, Japan; 5 Department of Plastic and Reconstructive Surgery, Saiseikai Utsunomiya Hospital, Tochigi, Japan; 6 Department of Plastic and Reconstructive Surgery, Nasu Red Cross Hospital, Nasu, Japan

**Keywords:** urinary incontinence, graciloplasty, pudendal nerve, microsurgery

## Abstract

An artificial sphincter implanted in the bulbous urethra to treat severe postprostatectomy urinary incontinence is effective, but embedding-associated complications can occur. We assessed the feasibility, efficacy, and safety of urethral graciloplasty cross-innervated by the pudendal nerve. A simulation surgery on three male fresh cadavers was performed. Both ends of the gracilis muscle were isolated only on its vascular pedicle with proximal end of the obturator nerve severed and transferred to the perineum. We examined whether the gracilis muscle could be wrapped around the bulbous urethra and whether the obturator nerve was long enough to suture with the pudendal nerve. In addition, surgery was performed on a 71-year-old male patient with severe urinary incontinence. The postoperative 12-month outcomes were assessed using a 24-hour pad test and urodynamic study. In all cadaveric simulations, the gracilis muscles could be wrapped around the bulbous urethra in a γ-loop configuration. The length of the obturator nerve was sufficient for neurorrhaphy with the pudendal nerve. In the clinical case, the postoperative course was uneventful. The mean maximum urethral closure pressure and functional profile length increased from 40.7 to 70 cm H _2_ O and from 40.1 to 45.3 mm, respectively. Although urinary incontinence was not completely cured, the patient was able to maintain urinary continence at night. Urethral graciloplasty cross-innervated by the pudendal nerve is effective in raising the urethral pressure and reducing urinary incontinence.

## Introduction

Male urinary incontinence is a common complication of radical prostatectomy (RP) with a significant impact on the patient's quality of life. For total urinary incontinence, an artificial urinary sphincter (AUS) is implanted in the bulbous urethra, and patients obtain urinary continence by manually adjusting the pressure. 1 2 Although effective, the device causes several complications, such as infections, erosion, mechanical failure, and urethral atrophy. The reoperation rate is 26.0% (15.0–45.0%). 2 Furthermore, the device is not physiological, because it takes several minutes to operate and there is no indication that the device will operate when cognitive function declines. The male sling procedure, synthetic mesh-like surgical tape is positioned around the bulbous urethra, is an alternative to AUS. This procedure is minimally invasive and has few complications, but its long-term data is not sufficient to prove whether it is as effective as the AUS. 3

The possibility of using the patient's own muscle flaps has been considered to reconstruct a neosphincter to overcome the limitations of the AUS procedure. In 1926, Deming 4 first reported graciloplasty to treat urinary incontinence. In 1956, Pickrell et al 5 reported six patients in whom the distal part of the gracilis muscle was transposed around the bladder neck and urethra; however, this procedure did not gain widespread clinical acceptance. Williams et al 6 and Janknegt et al 7 8 demonstrated dynamic graciloplasty, in which an electrical stimulating device was implanted to transform the gracilis muscle from a fatigue-prone to a fatigue-resistant muscle. However, clinical outcomes were disappointing because the urethra was wrapped around with the distal portion of the gracilis muscle, which was ischemic with a narrow width, without abundant contractile force, and sufficient volume. 8 9 Previously, we demonstrated the anatomic basis of anal sphincter reconstruction via dynamic graciloplasty with pudendal nerve anastomosis and verified the feasibility of this procedure. 10 Additionally, wrapping the urethra with a muscle flap increased both the maximum urethral closure pressure (MUCP) at rest, and electrical stimulation further increased the MUCP in a male rabbit model. 11 Guo et al 12 reported the efficacy of adynamic gracilis urethral myoplasty with a pedicled gracilis muscle flap wrapped around bulbar urethra for male patients with mild to moderate urinary incontinence. However, like the male sling, it is a static reconstruction and is ineffective for patients with severe incontinence. In this study, we proposed a new, more physiological procedure in which the well-vascularized belly at the middle part of the gracilis muscle is wrapped around the bulbous urethra and its motor nerve is sutured to the pudendal nerve, which originally controls the external urethral sphincter (EUS). We performed a simulation surgery on three fresh cadavers and a clinical study of a patient with severe postprostatectomy urinary incontinence in order to determine the feasibility of this procedure.

## Idea

The surgical procedure was simulated on three male cadavers at the Clinical Anatomy Laboratory at the Keio University School of Medicine, Tokyo, Japan, complying with the Guidelines for Cadaver Dissection in Education and Research of Clinical Medicine. The procedure was approved by the Japan Surgical Society and the Japanese Association of Anatomists.

### Surgical Procedure

**Fig. 1:**
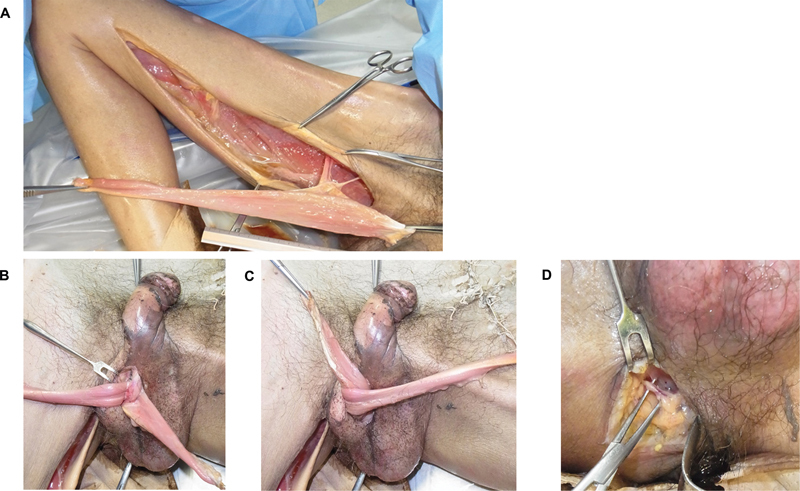
Cadaveric simulation of urethral gracioplasty reinnervated by the pudendal nerve. ( **A** ) In the lithotomy position, both origin and insertion of the gracilis muscle were severed, with preservation of the dominant vascular pedicle. The motor nerve of the muscle was dissected, and the proximal end was severed. ( **B** , **C** ) The proximal end of the gracilis muscle was subcutaneously tunneled into the perineum, passed under the bulbous urethra, folded back, and sutured to the ipsilateral inferior ramus of the pubis. Next, the distal end of the muscle was folded over its proximal side, achieving urethral graciloplasty in a γ-loop configuration. ( **D** ) Through an ipsilateral perineal incision, extrapelvic branches of the pudendal nerve with pudendal vessels were exposed and preserved for the donor nerve.

**Fig. 2:**
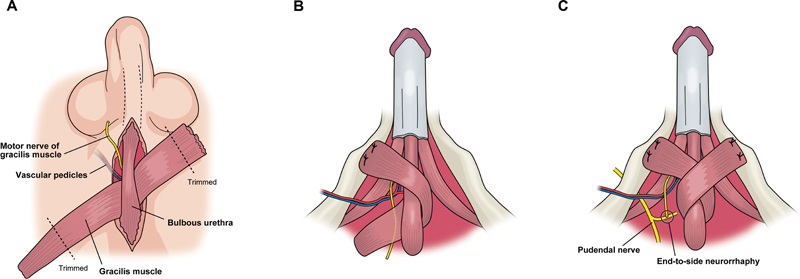
Operative schema. ( **A** ) The proximal end of the motor nerve into the gracilis muscle was severed, and the proximal end of the muscle was folded back under the bulbous urethra. ( **B** ) After the excess part at both ends of the gracilis muscle was trimmed, its proximal end was sutured to the ipsilateral inferior ramus of the pubis. ( **C** ) The distal end of the gracilis muscle was folded over its proximal side and sutured to the contralateral inferior ramus of the pubis.

The cadavers were positioned in the lithotomy position. A skin incision was made at the medial thigh, parallel to the long axis of the gracilis muscle. The origin of the muscle at the pubis was detached, and the dominant pedicle arising from the profunda femoris artery into its deep surface at the junction of the upper one-third with the lower two-thirds was preserved for the vascular pedicle ( Fig. 1A ). The motor nerve of the muscle was dissected proximally up to the bisection with the obturator nerve, and the proximal end was severed. If the nerve length was inadequate for subsequent nerve coaptation to the pudendal nerve without tension, it was further lengthened by dissecting distally into the muscle belly. 10 Both ends of the gracilis muscle were isolated only on its vascular pedicle, and the total muscle length, muscle width, distance from the point of the vascular pedicle into the gracilis muscle to the pubic symphysis, obturator nerve length, and diameter were measured. Through a perineal mid-incision, periurethral dissection was circumferentially performed at the level of the bulbous urethra, allowing urethral mobilization and muscle wrapping. After the excess parts at both the ends of the gracilis muscle were trimmed, its proximal end was subcutaneously tunneled without tension into the perineum, passed under the bulbous urethra, folded back, and sutured to the ipsilateral inferior ramus of the pubis ( Figs. 1B , 2A , and 2B ). Next, the distal end of the gracilis muscle was folded over its proximal side and sutured to the contralateral inferior ramus of the pubis ( Figs. 1C , 2A , and 2C ). Consequently, urethral graciloplasty was achieved in a γ-loop configuration, which could be used with the thicker and well-vascularized belly in the middle part of the gracilis muscle.

Somatic motor innervation of the EUS arises from the cell bodies of sacral spinal cord segments S2–S4 and is organized in Onuf's nucleus. The nerves to the EUS can be classified as (1) extrapelvic branches of the pudendal nerve, (2) intrapelvic branches of the pudendal nerve, or (3) branches of the inferior hypogastric plexus. 13 The extrapelvic branches travel through the greater sciatic notch to course anteriorly within Alcock's canal, then follow a constant course ventromedially, and several centimeters distally, give branches to the EUS and the superficial transverse perineal, bulbospongiosus, and ischiocavernosus muscles and to the scrotal and ventral penile skin. 13 14 Through an ipsilateral perineal incision, these extrapelvic branches with pudendal vessels were exposed at their entrance to Alcock's canal and preserved for the donor nerve ( Fig. 1D ). The gracilis muscle flap was subcutaneously tunneled into the perineal region, and we confirmed whether it could be sutured with the pudendal nerve, tension-free in an end-to-side fashion.

### Results

The gracilis muscle was easily transferred to the periurethral region by detaching both its origin and insertion and leaving only the vascular pedicle. The average length of the muscle belly was 291.7 (range 260–320) mm, and the average muscle width was 350 (range 330–370) mm ( Table 1 ). The vascular pedicle entered the muscle 76.7 (range 70–85) cm distal to the symphysis. After dissection and transection of the proximal end of the pudendal nerve at its bisection from the obturator nerve, its average length was 107.7 (range 95–123) mm and average diameter 2.5 (range 2–3) mm. The extrapelvic branch of the pudendal nerve was constantly confirmed, and its mean diameter was 2.7 (range 2.5–3.0) mm. In all simulations, the gracilis muscle was subcutaneously tunneled without tension into the perineum and could be wrapped around the bulbous urethra in a γ-loop configuration. The motor nerve of the muscles was sufficient for direct neurorrhaphy with the pudendal nerve, without requiring graft interposition.

**Table 1:** Cadaveric study: patient characteristics and surgical parameters

No.	Age (y)	Sex	Size of the gracilis muscle (mm)	Muscle length from the symphysis to vascular pedicle (mm)	Length of the obturator nerve (mm)	Diameter of the obturator nerve (mm)	Diameter of the pudendal nerve (mm)	The possibility of nerve coaptation
1	85	Male	37 × 260	70	95	3	2.5	Possible
2	88	Male	35 × 320	85	105	2.5	3	Possible
3	97	Male	33 × 295	75	123	2	2.5	Possible

## Case

A 71-year-old male patient presented with severe postprostatectomy urinary incontinence defined as a positive 24-hour pad test, which was 670 g (> 400 g). Physical therapy was ineffective, so surgery was indicated. The patient did not meet any of the following exclusion criteria: previous pelvic radiation therapy, incomplete bladder emptying, maximum flow urinary rate < 12 mL/s, detrusor overactivity, bladder capacity < 300 mL, and urethral stricture.

Urodynamic examinations were performed before surgery. The intraurethral pressure was measured three times using a pressure sensor unit (GMMS-600; Star Medical Co., Ltd., Tokyo, Japan) and a 14-French (Fr) pressure transducer (Unitip catheter; Unisensor AG, Attikon, Switzerland). The bladder was emptied. A catheter connected to the pressure sensor system was placed in the bladder 18 cm deep from the external meatus. The sensor was set to 0, and the catheter was withdrawn at 1 mm/s while recording the urethral pressure. The MUCP was defined as intrabladder pressure–urethral pressure and was set at 0 cm H _2_ O. Preoperative urodynamic examination revealed an MUCP of 40.7 cm H _2_ O and a functional profile length (FPL) of 40.1 mm ( Table 2 ).

**Table 2:** Comparison of pre- and postoperative urodynamic values

Variables	Preoperative values	Postoperative values
MUCP, mean (range)	46.1 ± 4.0 (42.5 − 50.5)	70 ± 2.29 (68 − 72.5)
FPL, mean (range)	40.1 ± 1.67 (38.8 − 42)	45.3 ± 1.59 (43.5 − 46.5)

Abbreviations: FPL, functional profile length (mm); MUCP, maximum urethral closure pressure (cm H _2_ O).

**Fig. 3:**
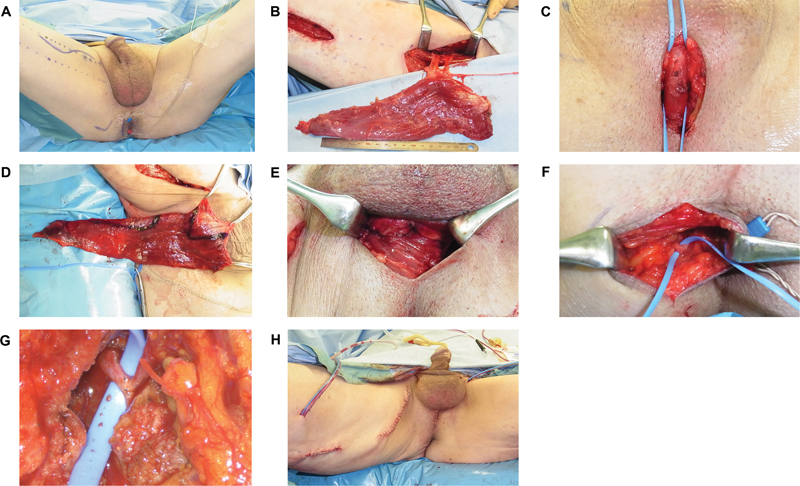
Surgical procedure used in the clinical case. ( **A** ) A preoperative incision line was made at the medial thigh and perineal region in the lithotomy position. ( **B** ) The origin and insertion of the gracilis muscle were detached, with preservation of the proximal dominant vascular pedicle. The motor nerve of the muscle was dissected, and the proximal end was severed. ( **C** ) The bulbous urethra was isolated and exposed circumferentially through a perineal mid-incision. ( **D** ) The gracilis muscle was subcutaneously tunneled into the perineum and trimmed to a width of 45 mm. ( **E** ) The gracilis muscle was wrapped around the bulbous urethra in a γ-loop configuration. ( **F** ) The pudendal nerve was bluntly dissected through the ipsilateral perineal incision. ( **G** ) The motor nerve of the gracilis muscle was subcutaneously tunneled into the perineal region and directly sutured with the pudendal nerve in an end-to-side fashion using a surgical microscope. ( **H** ) Image after completion of surgery.

The surgical procedure was performed under general anesthesia. Two skin incisions were made at the medial thigh, parallel to the long axis of the gracilis muscle in the lithotomy position ( Fig. 3A ). The origin of the muscle at the pubis was detached with a monopolar electric scalpel, and the proximal dominant vascular pedicle was preserved ( Fig. 3B ). The motor nerve of the muscle was dissected, and the proximal end was severed with a length of 12.5 cm. Next, the distal end of the muscle was detached, and minor vascular pedicles were ligated. The length of the muscle belly of the harvested muscle was 250 mm, and the maximal muscle width was 55 mm.

A 14 Fr Foley catheter was introduced into the bladder, and a perineal mid-incision was made. The bulbous urethra was isolated and exposed circumferentially ( Fig. 3C ), and the gracilis muscle was subcutaneously tunneled into the perineum, trimmed to a 45-mm width ( Fig. 3D ) and wrapped around the bulbous urethra in a γ-loop configuration ( Fig. 3E ).

After the excess parts at both the ends of the gracilis muscle were trimmed, the muscle was secured to each pubic ligament with horizontal mattress sutures, the tightening pressure of the muscle was adjusted to the extent that repeated urethral pressure reached 80 to 90 cm H _2_ O, and 400 mL of saline injected into the bladder was leaked by manual compression of the abdominal wall via intraoperative retrograde cystourethrography.

The pudendal nerve was bluntly dissected through the ipsilateral perineal incision ( Fig. 3F ). The motor nerve of the gracilis muscle was subcutaneously tunneled into the perineal region and directly sutured with the pudendal nerve, tension-free in an end-to-side fashion using a surgical microscope ( Fig. 3G ). Negative pressure drains were placed on the subcutaneous layer of the proximal thigh and perineal region, and the wound was closed ( Fig. 3H ).

The postoperative course was uneventful, without infection, muscle necrosis. The urinary catheter was removed 5 days after surgery, and no obvious urinary retention was observed. In order to convert to a new sphincter, rehabilitation was performed to tighten the urethral or anal sphincter.

**Fig. 4:**
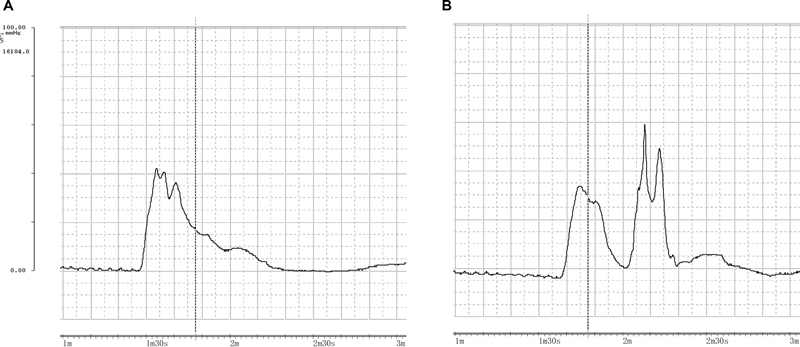
Pre- and postoperative urethral pressure profile. ( **A** ) Before surgery and ( **B** ) 12 months postoperatively.

The 24-hour pad test improved to 285 g (58% decrease) at 12 months postoperatively. On urodynamic examination, the mean MUCP and FPL were 70 cm H _2_ O and 45.3 mm, respectively ( Table 2 ). The preoperative urethral pressure profile showed a monomodal wave ( Fig. 4A ), consistent with the native EUS, while the postoperative profile showed a bimodal wave, with the second wave at the wrapped gracilis muscle ( Fig. 4B ). Although urinary incontinence was not completely cured, the patient could almost maintain urinary continence at night, leading to a reduction in the number of pads used.

## Discussion

Dysfunction of the EUS or urethral sphincter muscle is a major cause of urinary incontinence after RP. 15 16 Several studies showed a significant decrease of the MUCP and FPL related to urethral sphincteric function after RP. 17 In these studies, mean MUCP at baseline was 73 cm H _2_ O (range 49–95 cm H _2_ O) and mean MUCP after RP was 56 cm H _2_ O (range 30–83 cm H _2_ O). Mean FPL at baseline was 5.0 cm (range 4.3–6.1) and mean FPL after RP was 2.6 cm (range 1.6–3.1). Therefore, an accurate understanding of the urethral sphincter structure and innervation is important for sphincter reconstruction.

The EUS is located at the lower part of the urethra and forms a muscular coat ventral and lateral to the membranous urethra and prostate, the core of which is an omega-shaped loop around the membranous urethra. 18 Murakami et al 19 reported that the thick, lateral portion of the EUS retracts the urethra backward and upwards with the aid of the levator ani. In the elderly, sphincteric action is weak or incomplete, so the EUS maintains urinary continence by retracting the urethra backward and upwards with the aid of the levator sling, unlike the real sphincteric action in younger men. Burnet and Mostwin 20 described that urethral closure is achieved by the urethral sphincteric complex comprising the cylindrical EUS, ventral subpubic fascia, and medial fascia of the levator ani muscle. The action of the EUS is to draw the complex upwards and laterally along the inner undersurface of the pubis. Therefore, muscle fiber shortening at sphincteric contraction allows urinary continence, whereas elongation at sphincteric relaxation permits micturition.

In graciloplasty for fecal incontinence, various loops (e.g., γ, υ, c, α, and ε) can be used to wrap the gracilis muscle around the anus. 21 The classical γ-loop is often used, but it raises the issue of insufficient contraction because the distal part that is actually wrapped around the anus is thinner and has poor vascularity. Our modified γ-loop not only achieves sphincteric contraction but can also have the effect of lifting the urethra upwards by fixing both ends of the gracilis muscle to the pubic ligament, thereby resulting in a more physiological function. In addition, as the thicker and well-vascularized belly in the middle part of the gracilis muscle can be wrapped around the bulbous urethra, a new sphincter with a long FPL can be reconstructed.

The EUS predominantly consists of slow-twitch type I striated muscle, which is metabolically designed to provide sustained tone and contract slowly, whereas the levator ani consists of fast-twitch type II striated muscle, which contracts forcefully, rapidly, and for a short duration, 22 indicating that the periurethral striated musculature provides tonic contraction necessary for passive urinary continence, whereas the pelvic floor musculature provides rapid contraction necessary for voluntary interruption of the urinary stream. Martinez et al 23 reported that complete denervation of the EUS after bilateral pudendal nerve axotomy decreases the MUCP, which may cause urinary incontinence. Moreover, the increase in the MUCP via pudendal nerve end-to-end coaptation shows the primacy of the pudendal nerve in EUS innervation.

Research on cross-reinnervation shows that motor neurons influence the regulation of the mechanical and metabolic properties of skeletal muscles and mimic muscles. 22 23 24 25 26 A nerve to a slow-contracting muscle can convert a fast-contracting muscle almost completely to a slow type, and vice versa. Vukovich et al 22 demonstrated that the vascularized gracilis muscle flap wrapped around the rabbit urethra, cross-reinnervated by the pudendal nerve, has a reproducible response to bulbocavernosus reflex testing. Histologic findings of reinnervated gracilis muscle flaps suggest that reinnervation with the pudendal nerve facilitates a change in muscle myofiber physiology from fast twitch to slow twitch. Sato et al 27 demonstrated the feasibility of reconstructing perineal colostomy following anorectal resection using a transposed gluteus maximus muscle with pudendal nerve coaptation to achieve the physiological and histological characteristics of the external anal sphincter, such as voluntary contraction and relaxation in accordance with defecation signals from the nervous system, tonic activity in the resting state, and a receptor mechanism to sense the need to defecate, all of which are controlled by the pudendal nerve.

A recent experimental and clinical study proved the feasibility of transposition of skeletal muscle reinnervated by end-to-side neurorrhaphy with donor and recipient nerves. 28 29 End-to-side neurorrhaphy can induce collateral sprouting to the target muscle without losing donor nerve function. Pirro et al 30 demonstrated that anal sphincter reconstruction using the gracilis muscle transposed around the anal canal with end-to-side pudendal neurorrhaphy is anatomically achievable without losing the donor pudendal nerve function of the original anal sphincter and allows to integrate intact sensory mechanisms and obtain spontaneous relaxation with better muscular coordination during defecation. Sato et al 31 reported that the biceps femoris innervated by end-to-side pudendal nerve anastomosis contracted with the evoked potential in 5 of the 7 dogs (71%) and demonstrated electric activity at rest in 3 dogs (43%), which may be characteristics of the anal sphincter. In our case, by wrapping the gracilis muscle around the bulbous urethra and switching the obturator nerve to the pudendal nerve, both the MUCP and urinary incontinence improved, so we believe that the gracilis muscle was reinnervated by the pudendal nerve and that muscle fiber transformation occurred, resulting in tonic and voluntary contraction.

However, our procedure has some limitations. The reasons why daytime continence was not achieved despite the relatively high MUCP may include loosening of the transferred muscle fixation and denervation muscle atrophy until reinnervation by the pudendal nerve. The main differences between urinary and anal graciloplasty are that urine leakage occurs more easily than stool leakage, and the spiral-shaped configuration may cause twisting and pulling of the urethra rather than a circumferential closure around the bulbous urethra.

The AUS has the disadvantage of being difficult for the elderly to operate due to cognitive decline, whereas our procedure has the great advantage of restoring a more physiological urinary function. Further technical improvements such as the method of wrapping and securing the muscle are required to improve the treatment outcome.
